# Flexible and Emissivity-Adjustable Heat Flow Sensor Comprising a Carbon Nanotube/Polymer Composite

**DOI:** 10.3390/s25041243

**Published:** 2025-02-18

**Authors:** Kouji Suemori, Yuichiro Komatsu, Taiki Nobeshima

**Affiliations:** 1 Sensing System Research Center, National Institute of Advanced Industrial Science and Technology, 1-1-1 Higashi, Tsukuba 305-8565, Japan; 2 Human Augmentation Research Center, National Institute of Advanced Industrial Science and Technology, 6-2-3 Kashiwanoha, Kashiwa 277-0882, Japan

**Keywords:** heat flow sensor, flexible sensor, carbon nanotube, thermoelectric, emissivity

## Abstract

Heat flow sensors allow the straightforward measurement of the heat flow emitted from an object by attaching the sensor to the object. However, the inability of this method to control the surface emissivity of the sensor over a wide range lowers the measurement accuracy of a heat flow sensor. This study introduces a flexible heat flow sensor whose surface emissivity can be adjusted over a wide range. This sensor integrates a heat flow detector (HFD), which harnesses the Seebeck effect of a carbon nanotube/polymer composite to convert heat flow into voltage. This conversion exhibits a linear relationship with the heat flow, characterized by a proportional coefficient of 0.6556 mV·W^−1^. The sensor features an emissivity-controlling layer (ECL), comprising a thin Al film deposited on a flexible polymer film substrate. The surface emissivity of the layer can be adjusted between 0.06 and 0.88 by modulating the thickness of the Al coating. The ECL can be easily attached to and detached from the HFD. The proposed sensor enables the measurement of heat flow from heat sources with various emissivities by simply affixing it to the source. This study shows that the deployment of such sensors is useful for the advanced thermal management of diverse facilities.

## 1. Introduction

The heat flow emitted by objects reflects their status. Thus, heat flow sensing can be employed to monitor the conditions of various objects, such as humans [1,2] or various types of machinery [3]. Such heat flow sensing is potentially useful for the sophistication of cyber-physical systems [4,5,6,7,8,9,10] in industrial [7,8] and healthcare applications [9,10].

The estimation of the heat flow from an object to ambient air is traditionally conducted through finite-element calculations [11,12]. However, the acquisition of the necessary parameters for such calculations, such as the velocity of the airflow near the object and the precise dimensions of the object, is somewhat challenging. Thus, when such parameters cannot be obtained, a heat flow sensor that directly measures the heat flow from an object without requiring these parameters is useful. The heat flow sensor is used by attaching the sensor to the surface of the object to be measured. Given that the surfaces of many objects, including the human body and machinery, are typically curved, the sensor is required to be flexible. This flexibility ensures proper adherence to the contoured surfaces of such objects. The use of polymer-based thermoelectric materials, such as conducting polymers and composites made from carbon nanotubes (CNTs) and polymers, has been identified as a viable approach to creating flexible heat flow sensors [3,13]. For instance, our previous study demonstrated that a heat flow sensor fabricated using a CNT/polymethyl-methacrylate composite exhibited flexibility and high sensitivity that allowed the detection of minimal heat flows in the order of several tens of milliwatts [3].

The heat flow from an object to air encompasses convective and radiative components. For accurate measurements under steady state, a heat flow sensor must fulfill two conditions:

I. The thermal resistance of the sensor should be lower than that of the thermal boundary layer formed at the interface between the surface of the object and air.
II. The surface emissivity of the sensor should be close to that of the object to be measured.

When conditions I and II are satisfied, attaching the sensor to the object surface does not affect the heat flow emitted from the surface of the object. Thus, the heat flow measured using the sensor is close to the actual heat flow from the surface of the object. During heat flow measurements in air, condition I is probably satisfied by thinning the heat flow sensor because the thermal boundary layer of air has high thermal resistance [14]. However, it is challenging to satisfy condition II. To match the emissivities of the sensor and object surfaces, a facile method for controlling the emissivity of the sensor surface is required (Appendix A).

The emissivities of Al and most organic polymers are nearly zero and one, respectively. Thus, the emissivity of an Al coating formed on a polymer film is expected to widely vary with the Al thickness. Suppose the emissivity of the Al-polymer bilayer can be controlled by varying the Al thickness. In this case, the Al-polymer bilayer introduces a promising avenue for creating an emissivity-controlling layer (ECL) for heat flow sensors. Despite theoretical feasibility, the practical application of an Al-polymer bilayer as an ECL in heat flow sensing technology remains largely unexplored.

In this paper, we report on a heat flow sensor comprising a thin heat flow detector (HFD) and an Al/polyethyrene-naphthalate bilayer as the ECL. By varying the thickness of the Al layer, we successfully adjust the emissivity of the ECL within the range of 0.06–0.88. The flexibility of the ECL allows it to be conveniently attached to or detached from the HFD, aligning the surface emissivity of the sensor with that of the target object.

## 2. Materials and Methods

**Figure 1:**
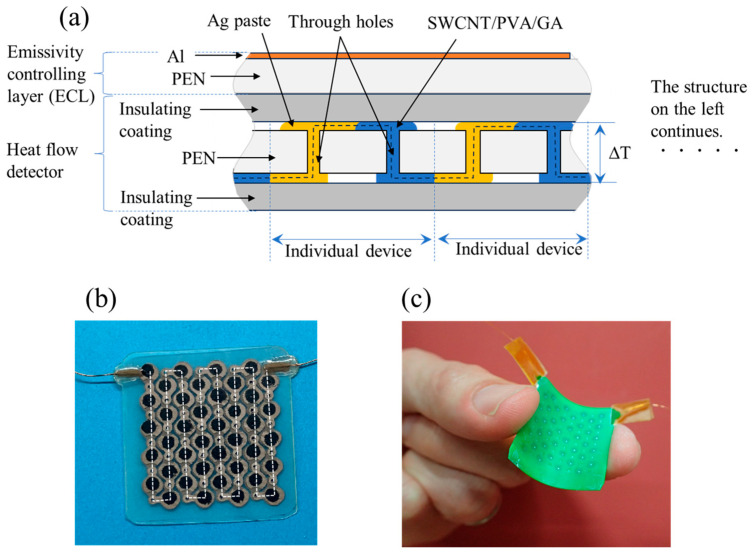
(**a**) Cross-sectional structure of heat flow sensor. The heat flow sensor comprises a bilayer of the heat flow detector (HFD) and emissivity-controlling layer (ECL). (**b**) Image of 40 individual devices formed on the PEN substrate. (**c**) Image of HFD.

We fabricated heat flow sensors; their cross-sectional structure is shown in Figure 1a. The sensor comprises an HFD and ECL. The HFD structure is similar to that described in our previous report [3]. The HFD converts the heat flow to voltage as follows: when heat flows along the out-of-plane direction of the HFD, a temperature difference is induced between the upper and lower sides of the HFD (Δ*T*, Figure 1a). This temperature difference is directly applied to the SWCNT/PVA/GA layer, which is a composite of single-walled CNT (SWCNT), polyvinyl alcohol (PVA), and gum arabic (GA). Therefore, voltage is generated between the upper and lower sides of the SWCNT/PVA/GA layer because of the Seebeck effect. The upper side of each SWCNT/PVA/GA layer was connected to the lower side of the next SWCNT/PVA/GA layer using the Ag paste. The black dotted line in Figure 1a indicates the electrical conducting pass. Hereinafter, we refer to a pair of single SWCNT/PVA/GA layer and Ag paste layer as an individual device (Figure 1a). Figure 1a shows two units of the individual devices; however, 40 individual devices were connected in series in actual devices. The voltage from HFD is the sum of the generated voltage in 40 SWCNT/PVA/GA layers.

The HFD had a polyethylene naphthalate (PEN) substrate film (thickness = 125 µm) with through holes (diameter = approximately 1 mm) (Figure 1a). The through holes in the PEN substrate were fabricated by laser cutter (Hajime, Oh-Laser Co., Ltd., Kawagoe, Japan) An aqueous solution of SWCNTs (0.2 wt%), PVA (0.1 wt%), and GA (1 wt%) was drop-cast onto the through holes of the substrate, which was followed by drying the solution on the hot plate at approximately 80 °C. Owing to the solution’s liquidity, the sidewalls of the through holes were coated with a film of the SWCNT/PVA/GA composite.

Because the sensitivity of HFD—the generated voltage per unit heat flux—is proportional to the number density of individual devices, a fine pattern of each layer is desired. Herein, we initially used an aqueous solution of SWCNT without PVA and GA—instead of a mixed solution of SWCNT, PVA, and GA—to fabricate HFD. However, fine pattering was difficult because the solution dropped on the substrate spread. Therefore, we added PVA and GA to the SWCNT solution to increase the viscosity, thereby suppressing the spreading of the solution on the substrate.

After fabricating SWCNT/PVA/GA layers, Ag paste was dispenser-printed on the substrate and through holes, followed by annealing at 130 °C for 30 min to evaporate the solvent of the Ag paste. Figure 1b depicts the PEN substrate after fabricating SWCNT/PVA/GA and Ag paste layers. The black and metallic parts are the SWCNT/PVA/GA composite and Ag paste layers, respectively. The 40 individual devices were connected along the white dotted line in Figure 1b. The thicknesses of the SWCNT/PVA/GA and Ag paste layers were approximately 12.6 and 7.7 µm, respectively. After fabricating the SWCNT/PVA/GA and Ag paste layers, the HFD was coated with an insulating layer (alkyd resin-based paint, Loihi Colr Neo, Sinloihi Co., Ltd., Kamakura, Japan) by dipping it into the alkyd resin-based paint dissolved in a thinner, followed by drying. Figure 1c shows an image of the HFD, exhibiting mechanical flexibility. The area and thickness of the HFD were 2.9 × 3.3 cm^2^, and 0.4 mm, respectively.

We measured the Seebeck coefficient of the SWCNT/PVA/GA layer using ZEM-3 (ADVANCE RIKO, Inc., Yokohama, Japan) under an Ar atmosphere. The width and length of the SWCNT/PVA/GA film used for the measurements were 15 and 1.5 mm, respectively.

**Figure 2:**
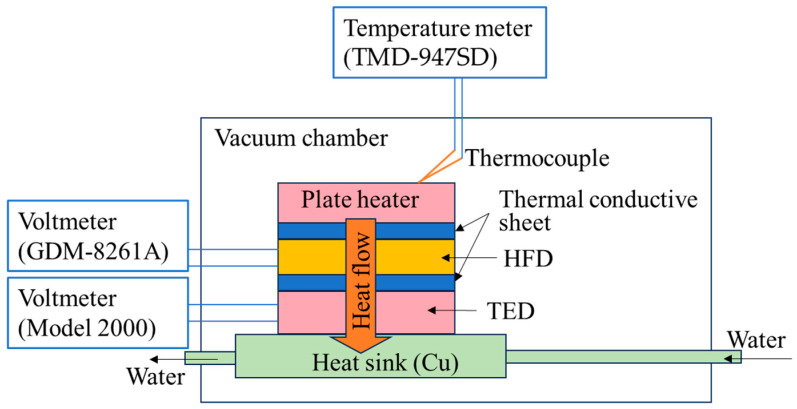
Scheme of equipment for measuring the relationship between the heat flow passing through the HFD and voltage. The HFD generates a voltage by a heat flow from the plate heater to the heat sink.

We measured the voltage–heat flow curve of the HFD using the equipment shown in Figure 2. The HFD was sandwiched between a plate heater and a thermoelectric device (TED). The temperature of the plate heater was measured using a K-type thermocouple with a line diameter of 50 μm, which was directly placed on top of the plate heater. The thermocouple was connected to a temperature meter (TMD-947SD, MOTHERTOOL Co., Ltd., Ueda, Japan), which has a reference junction compensation circuit. The heat flow generated by powering the plate heater passes through the HFD and TED and is absorbed by the heat sink. The heat flow caused voltage generation in the HFD and TED, which was measured using voltmeters (GDM-8261A, Good Will Instrument Co., Ltd., New Taipei, Taiwan, and Model 2000, Keithley Instruments Inc., Cleveland, OH, USA) connected to each of the HFD and TED. The area of the heater and TED was approximately the same as that of the HFD. To avoid heat leakage from the equipment into ambient air via convection, the measurement system (other than voltmeters and temperature meter) was placed in a vacuum chamber with a vacuum of less than 1 × 10^−2^ Torr. We neglected the heat leakage by radiation from the side area of the HFD and thermal conductive sheets because it was calculated to be less than 0.1% of the total heat flow (Appendix B). Under this condition, heat flow (*q*) passing through the TED is the same as that passing through the HFD. *q* was obtained by $q=A\cdot V_{TED}$, where *A* is the proportional coefficient and *V_TED_* is the voltage generated by the TED. Therefore, *q* can be obtained by measuring *V_TED_* using a voltmeter (Model 2000, Keithley Instruments Inc.); *A* is calculated as $A=1/\left(R\cdot S\right)$, where *R* and *S* are the thermal resistance and Seebeck coefficient of TED, respectively. *R* and *S*, obtained from tests commissioned to ADVANCE RIKO Inc., were 2.33 °C/W and 27.2 mV/°C, respectively. We measured the voltage (*V*) generated by the HFD with various *q* values to estimate the relationship between the *q* and *V* for HFD.

**Figure 3:**
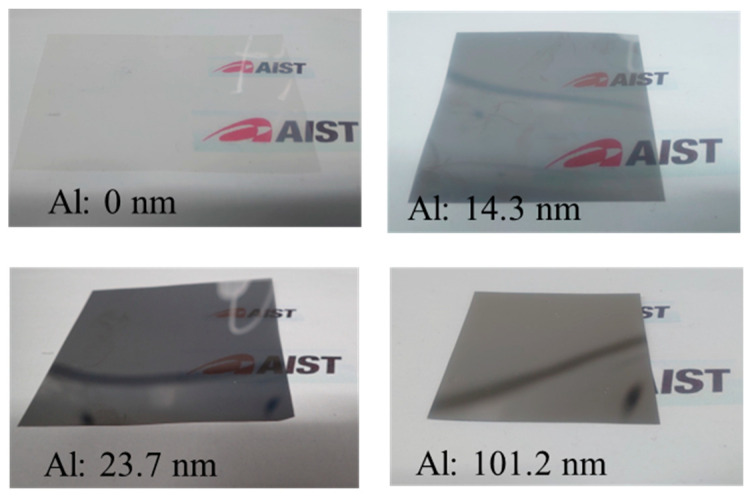
Images of ECLs with Al thicknesses of 0, 14.3, 23.7, and 101.4 nm.

The ECL was fabricated via thermal Al evaporation on the PEN film (thickness = 75 μm) under a vacuum of less than 1 × 10^−3^ Pa. Figure 3 shows images of the ECL, which was optically semitransparent when the Al thickness was low. The emissivity of the ECL surface was measured using an emissivity meter (TSS-5X-3; Japan Sensor Corp., Minatoku, Japan). The structure of the ECL surface was examined via atomic force microscopy (AFM, Bruker Japan K.K., Yokohama, Japan). Finally, to complete the fabrication of the heat flow sensor, the ECL was adhered to the HFD surface using a thermally conductive paste.

## 3. Results and Discussion

### 3.1. Heat Flow Detector

**Figure 4:**
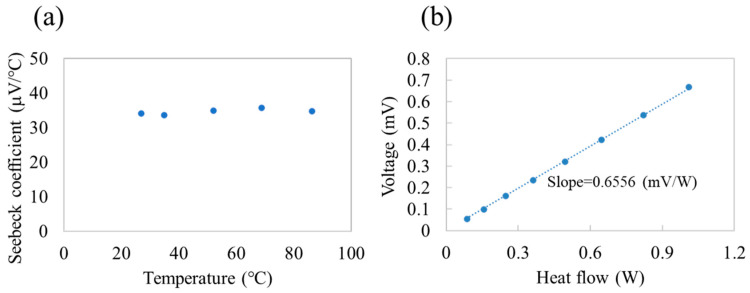
(**a**) Variation in the Seebeck coefficient with temperature for a SWCNT/PVA/GA film formed on a PEN substrate. (**b**) Variation in voltage with heat flow for the heat flow detector.

The voltage generation capability of HFD depends on the Seebeck effect of the SWCNT/PVA/GA layers. Thus, we measured the Seebeck coefficient of the SWCNT/PVA/GA layer (Figure 4a), which was approximately 35 μV/°C from room temperature to 100 °C; this value is similar to that of various composites comprising SWCNT and insulating polymer [15,16,17]. Figure 4b shows the voltage (*V*) as a function of *q* passing through the HFD. The relationship between *q* and *V* in the HFD was practically linear with a proportional constant of approximately 0.6556 mV·W^−1^ (Figure 4b), i.e.,

(1) $$q=\frac{V}{0.0006556}.$$

Thus, the heat *q* passing through the HFD can be estimated by measuring the voltage *V*. The sensor sensitivity is typically evaluated by the voltage generated by the unit heat flux, i.e., the heat flow per unit area; thus, the unit of sensitivity is V/(W·m^−2^). The sensitivity of our HFD calculated using 0.65566 mV·W^−1^ and the area of sensor 9.57 × 10^−4^ m^2^ was 0.63 μV/(W·m^−2^). We compared this value with that of reported heat flow sensors. Li et al. reported a sensitivity of 0.06193 μV/(W·m^−2^) for a heat flow sensor comprising ITO/In_2_O_3_ thermopile on a Ni alloy [18]. Chen et al. developed a heat flow sensor based on the transverse thermoelectric effect of YBa2Cu3O7-δ with a sensitivity of 0.023 μV/(W·m^−2^) [19]. Wang et al. fabricated a thermopile-type heat flow sensor with a sensitivity of 0.267 μV/(W·m^−2^) using the printed circuit board process [20]. Li et al. developed a heat flow sensor comprising a fine array of ITO/In_2_O_3_ thermopile and reported a sensitivity of 13.43 μV/(W·m^−2^) [21]. Thus, despite using an inherently flexible material, i.e., SWCNT/PVA/GA, the sensitivity of our HFD is comparable to that of reported heat flow sensors.

We estimated the signal-to-noise ratio (SNR) as follows [22]:

(2) $$SNR=20log\frac{V}{U_{J}},$$

where *U_J_* is Johnson noise. Johnson noise is generated by Brownian motion of free electrons and is expressed as

(3) $$U_{J}=\sqrt{4\cdot k_{b}{\cdot T_{s}\cdot R}_{s}\cdot ∆f},$$

where *k_b_*, *R_s_*, *Ts*, and Δ*f* are the Boltzmann constant, resistance of the HFD, temperature of HFD, and frequency bandwidth, respectively. The *Rs* of the HFD was 17.15 kΩ. Δ*f* is obtained by the integration period of the A–D converter in the voltmeter and probably depends on the model and manufacturer of the voltmeter. However, typical Δ*f* is in the order of 10 Hz [22]. Using Δ*f* = 10 Hz and *Ts* = 25 °C, *U_J_* was calculated to be 5.31 × 10^−8^ V. *V* observed in Figure 4b was 0.0531–0.668 mV. SNR was calculated using Equation (2), and the value was 60–82 dB, which corresponds to *V*/*U_J_* values of 1.0 × 10^3^ to 1.3 × 10^4^.

### 3.2. Heat Flow Sensor with Emissivity-Controlling Layer

**Figure 5:**
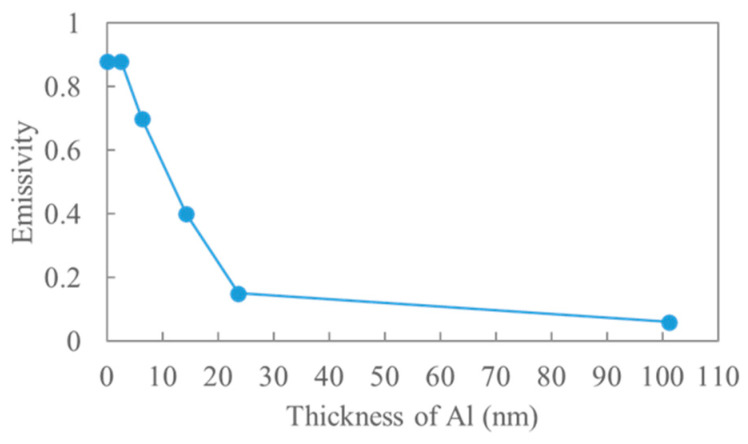
Emissivity of the ECL as a function of the Al thickness.

Figure 5 shows the emissivity of the ECL surface as a function of the Al thickness. The emissivity monotonically decreased with an increase in the Al thickness. The emissivity ranged between 0.06 (Al thickness = 101.2 nm) and 0.88 (Al thickness = 0 nm). The decrease in emissivity with an increase in the Al thickness was practically complete at an Al thickness of 25 nm. The Al thickness can be controlled with the accuracy of the order of Å using the thermal evaporation technique, which is sufficiently accurate for controlling the Al thickness within a range of 0–25 nm. Thus, the emissivity of the ECL surface can be controlled over a wide range by controlling the Al thickness.

**Figure 6:**
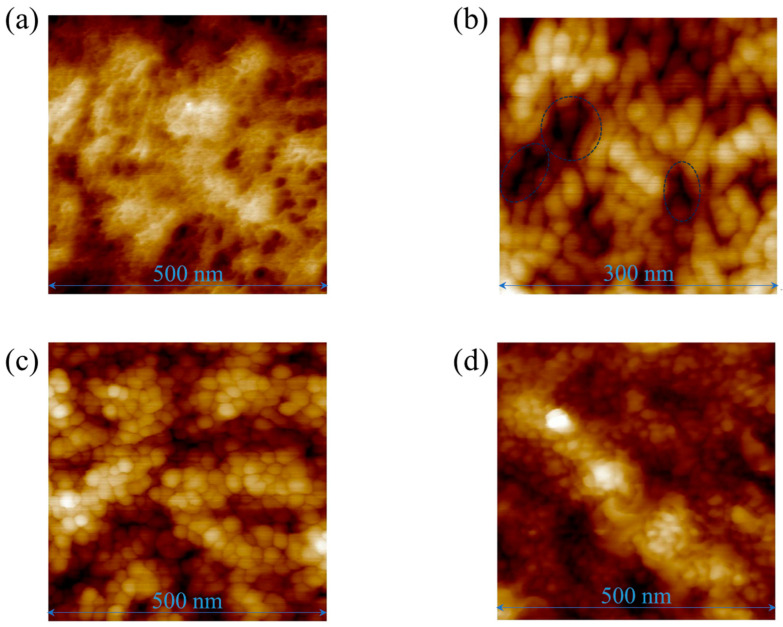
AFM images of the ECL surface with Al thicknesses of (**a**) 0, (**b**) 2.5, (**c**) 6, and (**d**) 14.3 nm.

Figure 6a–d show AFM images of the ECL surface with Al thicknesses of 0, 2.5, 6, and 14.3 nm, respectively. The Al-formed grains, which are generally observed structures [23,24,25]. The diameter of grains was several tens of nanometers. When the Al thickness was 2.5 nm, a small part was not covered by Al (enclosed area, Figure 6b), whereas Al covered most parts. The PEN surface was covered almost entirely by Al at a thickness of 6 nm (Figure 6c). However, the emissivity of the ECL with 6 nm-thick Al was 0.72, indicating that most of the electromagnetic waves generated by molecular vibration in the PEN passed through the Al layer because the Al was thin. The shielding of the electromagnetic waves inevitably increased with an increase in the Al thickness, which decreased the emissivity (Figure 5).

**Figure 7:**
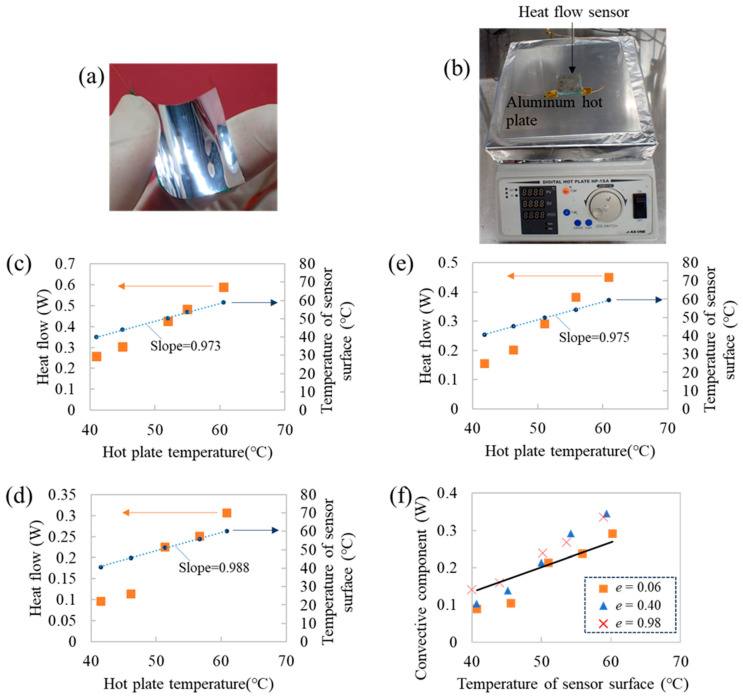
Measurement of heat flow from the hot plate under natural convection. (**a**) Image of a heat flow sensor whose surface has an ECL. (**b**) Heat flow sensor attached to an Al hot plate. (**c**–**e**) Heat flow (left axis) and surface temperature of heat flow sensor (right axis) as functions of the hot plate temperature for sensors without an ECL (**c**), with ECLs (*e* = 0.06 (**d**) and *e* = 0.4 (**e**)). The sensor surface temperatures were fitted by a line with slopes of 0.973 (**c**), 0.988 (**d**), and 0.975 (**e**). (**f**) Convection component of heat flow for each sensor as a function of the sensor surface temperature. The squares, triangles, and crosses denote the data obtained using the sensor with 0.06-ECL, 0.4-ECL, and without ECL, respectively. The solid line indicates the calculated convection heat transfer using the 1st term of Equation (4) with *h* = 7 W/m^2^ °C and Tair = 19 °C.

We fabricated a heat flow sensor by attaching the ECL to the HFD (Figure 7a). Generally, the heat flow emitted from a heat source to ambient air is described by

(4) $$q=A\cdot h\left(T-T_{air}\right)+A\cdot e\cdot \sigma \left(T^{4}-T_{air}^{4}\right),$$

where *A*, *h*, *e*, *T*, *T_air_*, and σ denote the area of the heat source, heat-transfer coefficient of air, emissivity of the object surface, surface temperature of the object, ambient air temperature, and Stefan–Boltzmann constant, respectively [26,27,28]. The first and second terms on the right side of Equation (4) denote convection and radiation, respectively. Here, the heat flow sensor attached to the object surface must exhibit the same heat flow as that emitted from the object surface in the absence of the sensor. The sensor attached to the surface of the object imitates the shape of the surface of the object because the sensor is thin and flexible. Thus, the changes in *A* and *h* achieved by attaching the sensor are, in principle, small. Equation (4) indicates the following matter: when the *T* and *e* of the sensor surface are the same as those of the object surface, the heat flow emitted from the sensor surface mirrors that from the object surface. Here, the *e* of the sensor surface can be aligned with that of the object surface by attaching an ECL with the same *e* to the object surface on the HFD. Thus, aligning the surface temperature of the sensor to that of the object, i.e., the sensor satisfying condition I, is necessary and adequate to correctly measure the heat flow from an object.

We conducted the following experiment to confirm that the heat flow sensor satisfied condition I. We attached the heat flow sensor to a hot plate made of Al (Figure 7b) and measured the surface temperature of the sensor, i.e., a surface temperature of ECL (right axis of Figure 7c–e). The relationships between the sensor surface and hot plate temperatures were fitted by the line with a slope of almost 1, indicating that the temperature of the sensor surface was almost the same as the hot plate surface temperature. Thus, the heat flow sensor satisfied condition I. The sensor mainly comprised an organic polymer, with a typical thermal conductivity of 0.1 W/m°C, and a small amount of Ag paste and CNT, whose thermal conductivity exceeds that of organic polymers. Thus, the thermal resistance per unit area of the sensor, which is calculated by the thickness divided by thermal conductivity, was estimated to be less than 5.25 × 10^−3^ °C/(W/m^2^). This was two orders of magnitude lower than the thermal resistance of the thermal boundary layer of air [14]. Thus, the sensor surface temperature was almost the same as the hot plate surface temperature. The HFD shown in Figure 1a can detect a minute temperature difference in the order of 10^−3^ °C formed in the HFD [3]. Thus, the sensor can measure the heat flow even when the temperature difference formed in the sensor is small.

### 3.3. Demonstration of the Heat Flow Sensor

The ECLs were easily attached to and detached from the HFD using a thermal conductive paste. Therefore, Figure 1a denotes that the sensor structure has the ease of surface emissivity, which can be adjusted by replacing the ECL with one that exhibits the desired emissivity, thereby allowing the analysis of heat flow accompanied by heat transfer from the surface of an object to air.

Finite-element calculations are frequently employed to estimate heat transfer phenomena. When heat is transferred through the air/object interface, the finite element calculation requires the *h* of air in advance. Here, we showed that heat flow sensors can be used to estimate the *h* of air as follows. We attached a heat flow sensor without an ECL to the hot plate, which was almost the same arrangement as that in Figure 7b, and measured the heat flow using the sensor (squares, Figure 7c). The HFD functions as a heat flow sensor with the surface *e* of 0.98 because the *e* of the surface coating of the HFD was 0.98. Thereafter, we attached an ECL with an *e* of 0.06 (0.06-ECL) to the HFD and subsequently measured the *q* using the heat flow sensor (squares, Figure 7d). The 0.06-ECL surface exhibited almost no radiation owing to its low *e*; the 0.06-ECL-attached heat flow sensor exhibited a lower *q* than the heat flow sensor without an ECL. We replaced the 0.06-ECL on the HFD with the 0.4-ECL and measured the heat flow (squares, Figure 7e); an intermediate heat flow was observed without an ECL and with the 0.06-ECL.

The amount of radiation heat transfer from the sensor surface can be calculated using the 2nd term of Equation (4), where *T* is the temperature of the sensor surface. We extracted the convection component of heat flow by subtracting the radiation component from the measured heat flow for sensors with various *e* values (Figure 7f). The amount of convection for each sensor was almost the same and was fitted by the 1st term of Equation (4) with the *h* of 7 W/m^2^ °C (bold line, Figure 7f), i.e., the *h* was estimated at 7 W/m^2^ °C. It is well known that the *h* of air is dependent on the airflow velocity (*v*) and roughly estimated by *h* = 3.8*v* + 5.7 [W°C^−1^m^−2^] [14]. Thus, when the airflow velocity is unknown, it is difficult to estimate the *h* of air in the actual environment. This result indicates that the *h* of air can be estimated by measuring the heat flow using sensors with various *e* values even when *v* is unknown.

**Figure 8:**
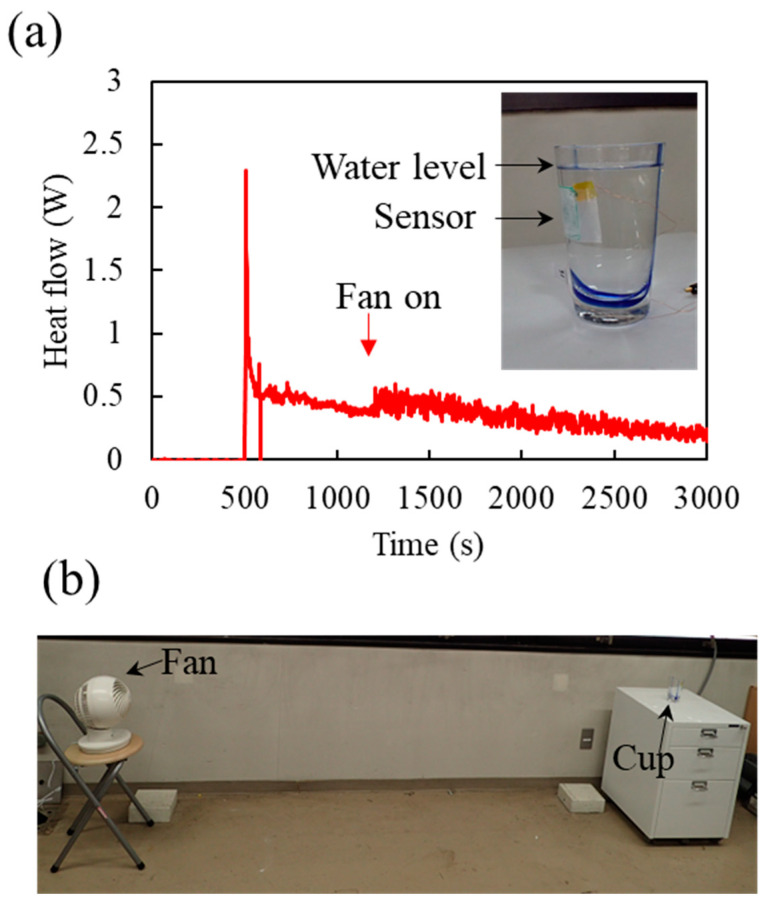
Measurement of heat flow from a glass cup filled with hot water. (**a**) Heat flow from the glass cup as a function of time. Hot water (temperature = approximately 60 °C) was poured into the cup at 500 s. The inset shows the water level and heat flow sensor. An electric fan placed 2.5 m from the cup was switched on at 1190 s (downward arrow) to generate wind near the cup. (**b**) Image of electric fan and cup. The height of the electric fan was almost the same as that of the cup.

The heat flow sensor allows the facile measurement of heat emission from object surfaces even when the heat emission process is under transient conditions. We measured the heat flow emitted from a glass cup filled with hot water (Figure 8a). The glass surface is known to have an *e* of approximately 0.9 [29], which is practically the same as that of an ECL with an Al thickness of 0 nm (Figure 5). Thus, we placed the ECL with an Al thickness of 0 nm on the HFD. We poured hot water with a temperature of approximately 60 °C at 500 s. The heat flow rapidly increased immediately after the hot water was poured, followed by a rapid decrease over time (Figure 8a). The hot water caused a heat flow from the water to the air, which rapidly increased the heat flow at 500 s. The heat flow into the air caused an increase in the air temperature at the boundary of the air and heat flow sensor surface. This rapidly decreased the heat flow with time. This rapid decrease was practically complete at approximately 600 s, indicating that the completion of the thermal boundary layer formation took approximately 100 s. We brewed wind into the cup at 1190 s (downward arrow, Figure 8a) by switching on an electric fan placed approximately 2.5 m from the cup (Figure 8b). The heat flow increased because of the increase in *h* caused by the increase in airflow velocity.

The heat flow sensor exhibited mechanical flexibility because all the components used for fabricating the sensor were flexible (Figure 1c and Figure 7a). Thus, the heat flow sensor can be attached to a curved heat source surface, such as a cup surface (Figure 8a). The heat flow sensors are useful for the facile measurement of heat flow from various objects, as demonstrated above.

## 4. Conclusions

Here, we introduced a design of flexible heat flow sensors incorporating Al-PEN bilayers as ECLs. The emissivity of the ECL surface was controlled over a wide range (0.06–0.88). Thus, the proposed heat flow sensor can detect the heat flow from objects with various surface emissivities. In addition, the proposed heat flow sensor is flexible, allowing the measurement of heat flow from a nonflat surface. The experiments demonstrate that these sensors can measure the heat flow by directly applying the sensor to the surface of the object. Such sensors are probably useful for the convenient estimation of the heat flow emitted from an object.

## Figures and Tables

**Figure A1:**
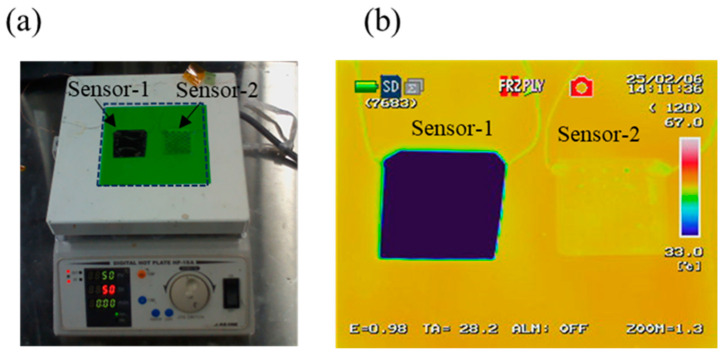
Photograph (**a**) and infrared image (**b**) of two sensors placed on a hot plate at a temperature of 50 °C.

**Figure A2:**
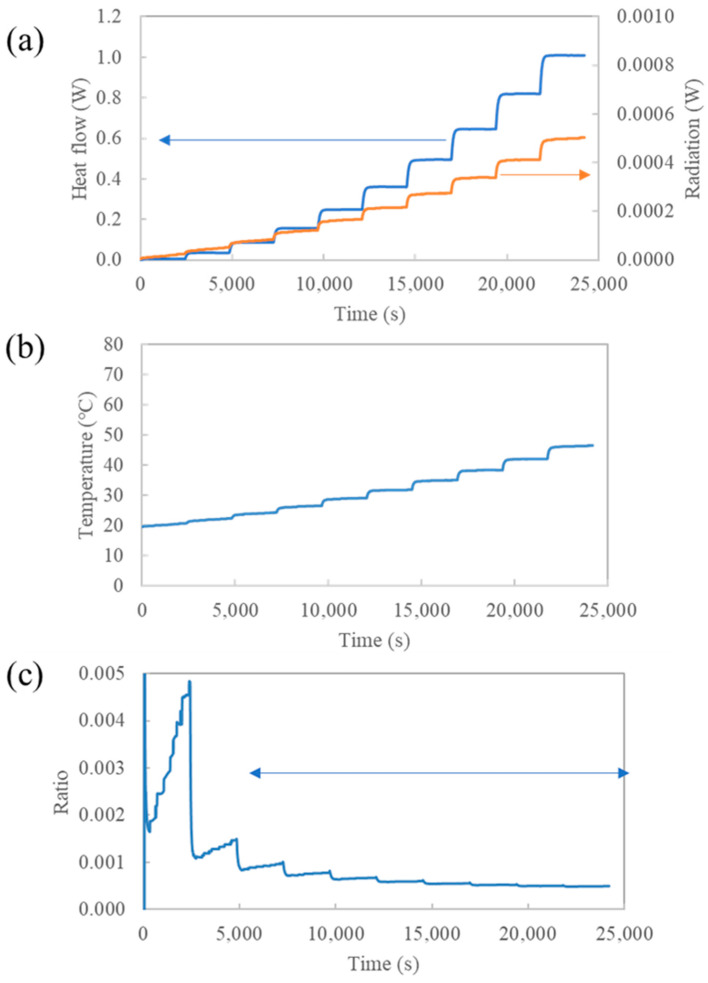
Radiation from the side surface of the heat flow detector (HFD) and thermal conductive sheets. (**a**) heat flow as a function of time. We increased the power input to the heater every 2400 s. (**b**) Temperature of the plate heater surface as a function of time. (**c**) Ratio of heat flow (left axis of a) and $\sigma \cdot SA\left({Th}^{4}-{Ta}^{4}\right)$.

## Data Availability

The original contributions presented in this study are included in the article. Further inquiries can be directed to the corresponding author.

## Abbreviations

The following abbreviations are used in this manuscript:

HFD	Heat flow detector
ECL	Emissivity-controlling layer
CNT	Carbon nanotube
PEN	Polyethylene naphthalate
GA	Gum Arabic
TED	Thermoelectric device

## Appendix A

Figure A1 shows a photograph (Figure A1a) and an infrared camera image (Figure A1b) of the two sensors attached to a hot plate. The green part of the hot plate surface (enclosed by a dotted line in Figure A1a) exhibited a surface emissivity of 0.98. The surface of sensor-1 was coated with Al (emissivity = 0.06), and that of sensor-2 was coated with organic paint (emissivity = 0.98). In Fig. A1b, the surface of sensor-2 was almost the same color as that of the hot plate, whereas sensor-1 was dark, indicating that radiation from the surface of sensor-1 was lower than that from the surfaces of sensor-2 and the hot plate. Therefore, controlling the emissivity of the sensor surface is indispensable in measuring heat flow using a heat flow sensor.

## Appendix B

During the measurement using equipment in Figure 2, radiation from the side surface of the HFD and thermal conductive sheets is regarded as heat leakage that lowers the measurement accuracy for HFD characteristics. We estimated the amount of radiant heat from the side surface and concluded that the heat leakage by radiation was negligible, as shown below. To eliminate the heat leakage due to convection, the measurement chamber of Figure 2 was kept under a vacuum of <1 × 10^−2^ Torr. The heat flow depicted in Figure 2 is formed by the electric power input into the plate heater. Figure A2a is the heat flow as a function of time. We increased the power input to the heater every 2400 s. The heat flow increased with an increase in the power input to the heater. During the measurements, we monitored the temperature of the top of the plate heater, which was increased (Figure A2b). The temperature gradient has the direction from the plate heater to the heat sink. Thus, the temperature of the side surface of the HFD and thermal conductive sheets was lower than that of the plate heater. In this case, the total amount of radiation from the side surface of the HFD and thermal conductive sheets (*qe*) can be described as $qe<\sigma \cdot SA\left({Th}^{4}-{Ta}^{4}\right)$, where *σ*, *SA*, *Th*, and *Ta* are the Stefan–Boltzmann constant, sum of the area of the side surface of the HFD and thermal conductive sheets, temperature of the plate heater, and temperature of the object around the equipment, primarily comprising the wall of the vacuum chamber. The right axis of Figure A2a is the calculated value of $\sigma \cdot SA\left({Th}^{4}-{Ta}^{4}\right)$, where *Th* and *Ta* are the measured plate heater temperature (Figure A2b) and room temperature (19 °C) during the measurement, respectively. Figure A2c shows the ratio of heat flow (left axis of Figure A2a) and $\sigma \cdot SA\left({Th}^{4}-{Ta}^{4}\right)$. We used data points obtained at over 5000 s, the region depicted by the arrow in Figure A2c, to measure the heat flow sensor characteristics. In this region, the ratio was <0.001, indicating that *qe* was <0.1% of the heat flow passing through the HFD. Thus, we neglected radiation from the side surface of the HFD and thermal conductive sheets.
